# Attenuation of antigen-specific T helper 1 immunity by *Neolitsea hiiranensis* and its derived terpenoids

**DOI:** 10.7717/peerj.2758

**Published:** 2016-12-07

**Authors:** Yin-Hua Cheng, Ih-Sheng Chen, Ying-Chi Lin, Chun-Wei Tung, Hsun-Shuo Chang, Chia-Chi Wang

**Affiliations:** 1 Ph.D. Program in Toxicology, College of Pharmacy, Kaohsiung Medical University, Kaohsiung, Taiwan; 2 School of Pharmacy, Kaohsiung Medical University, Kaohsiung, Taiwan

**Keywords:** T-bet, Neolitsea, IFN-γ, Th1 cells, Terpenoids, β-caryophyllene oxide

## Abstract

**Background:**

T cells play a pivotal role in the adaptive immunity that participates in a wide range of immune responses through a complicated cytokine network. Imbalance of T-cell responses is involved in several immune disorders. *Neolitsea* species, one of the biggest genera in the family Lauraceae, have been employed widely as folk medicines for a long time in Asia. Previous phytochemical investigations revealed the abundance of terpenes in the leaves of *N. hiiranensis*, an endemic *Neolitsea* in Taiwan, and demonstrated anti-inflammatory activities. However, the effect of *N. hiiranensis* on the functionality of immune cells, especially T cells, is still unclear. In this study, we utilize *in vitro* and *in vivo* approaches to characterize the effects of leaves of *N. hiiranensis* and its terpenoids on adaptive immune responses.

**Methods:**

Dried leaves of *N. hiiranensis* were extracted three times with cold methanol to prepare crude extracts and to isolate its secondary metabolites. The ovalbumin (OVA)-sensitized BALB/c mice were administrated with *N. hiiranensis* extracts (5–20 mg/kg). The serum and splenocytes of treated mice were collected to evaluate the immunomodulatory effects of *N. hiiranensis* on the production of OVA-specific antibodies and cytokines. To further identify the *N. hiiranensis-*derived compounds with immunomodulatory potentials, OVA-primed splenocytes were treated with compounds isolated from *N. hiiranensis* by determining the cell viability, cytokine productions, and mRNA expression in the presence of OVA *in vitro*.

**Results:**

Crude extracts of leaves of *N. hiiranensis* significantly inhibited IL-12, IFN-*γ*, and IL-2 cytokine productions as well as the serum levels of antigen-specific IgM and IgG_2a_ *in vivo*. Two of fourteen selected terpenoids and one diterpenoid derived from the leaves of *N. hiiranensis* suppressed IFN-*γ in vitro*. In addition, *β*-caryophyllene oxide attenuated the expression of IFN-*γ*, T-bet, and IL-12R*β*2 in a dose-dependent manner. *N. hiiranensis-*derived *β*-caryophyllene oxide inhibited several aspects of adaptive immune responses, including T-cell differentiation, IFN-*γ* production, and Th1-assocaited genes.

**Conclusion:**

As IFN-*γ* is the key cytokine secreted by T helper-1 cells and plays a pivotal role in Th1 immune responses, our results suggested that the *N. hiiranensis* and its terpenoids may possess potential therapeutic effects on Th1-mediated immune disorders.

## Introduction

T helper (Th) cells play a pivotal role in our immune system against environmental stimulations. They participated in a wide range of immune responses via cell–cell interaction with other cells through a complicated cytokine network. Th1 cells, producing interferon-gamma (IFN-*γ*), interleukin-2 (IL-2) in the regulation of cellular immunity. On the other side, Th2 cells promoted humoral immunity via secreting IL-4, IL-5 and IL-13 (Lazarevic & Glimcher, 2011; Lin & Lin, 2011). Abnormal immunostimulation and the imbalance of Th1/Th2 responses may lead to a variety of immune diseases. For example, the dominant of Th1 cells is associated with multiple sclerosis, Crohn’s disease, rheumatoid arthritis, and delayed type hypersensitivity (DTH) (Agnello et al., 2003; Lazarevic & Glimcher, 2011).

The immunosuppressive drugs, such as glucocorticoids, cyclosporine A, infliximab, and etanercept, were developed to treat the over-reactive immune responses, inflammation or T-cell mediated immune disorders (Diluvio et al., 2010; Lee et al., 2002; Lin & Wang, 2016; Rezzani, 2004; Rezzani, 2006; Srivastava, Alexander & Tuthill, 2005; Tan et al., 2007; Torres et al., 2011; Weinberg et al., 2005; Wolff, McKay & Brugarolas, 2014; Zeevi et al., 1987). However, severe adverse effects have been associated with the long-term usage of these immunosuppressants. To discover new botanicals with differential immunomodulatory effects on T-cell function may provide more therapeutics for different T-cell-mediated immune disorders. Many natural compounds isolated from medicinal plants have been shown to possess therapeutic potentials for Th1-associated diseases (Jeon et al., 2015; Wu et al., 2014).

*Neolitsea* is one of the major genera in Lauraceae family. There are about 85 species in Asiatic and Malaysia, including six endemic species in Taiwan (Liao, 1996; Liou et al., 2011). These evergreen shrubs or trees have long been used as traditional folk medicines to treat carcinomatous swelling, abdominal pain, diarrhea, rheumatism, nausea and vomiting (Xie, 1996). These plants contain various bioactive components including sesquiterpenes which are known to have anti-inflammatory effects (Chang et al., 2002; Chen et al., 2005) and terpenoids which have been demonstrated to possess immunomodulatory effects on LPS-stimulated splenocytes *in vitro* (Ku & Lin, 2013).

*N. hiiranensis* is an endemic *Neolitsea* in Taiwan containing a rich amount of sesquiterpenoids which have been documented to possess anti-inflammation activity (Liou et al., 2011; Wu & Li, 1995). Hiiranlactone B and hiiranlactone D, the sesquiterpenoids isolated from the leaves of *N. hiiranensis,* suppressed the *N*-formyl-methionyl-leucyl-phenylalanine (fMLP)-induced generation of the superoxide anion by human neutrophils (Ho et al., 2011; Liou et al., 2011). Pseudoneolinderane and isolinderalactone isolated from the roots of *N. hiiranensis* have been shown their anti-inflammatory activities (Wu & Li, 1995). These data indicated the potential immunomodulatory effects of *N. hiiranensis* on innate immune responses. However, the effect of *N. hiiranensis* on T-cell functionality remains unclear.

In this study, we first determined the effects of *N. hiiranensis* on antigenspecific T cells *in vivo*. We then selected fourteen secondary metabolites with none/less hepatotoxicity and genotoxicity from leaves of *N*. *hiiranensis* to further investigate the potential immunomodulatory effects of the therapeutic botanicals for Th1 immune disorders. We report here that the administration of *N*. *hiiranensis* didn’t affect body weight, spleen index, and spleen cellularity *in vivo*. Several antigen-specific immune responses were attenuated by *N. hiiranensis* and its terpenoids.

## Materials and Methods

### Extraction and isolation from the Taiwanese N. hiiranensis

The crude extracts and the secondary metabolites were prepared and isolated from the leaves of *N. hiiranensis* according to the previous report (Liou et al., 2011). Briefly, Taiwanese *N. hiiranensis* were collected at Mudan (Pingtung County, Taiwan) and identified by Dr. Ih-Sheng Chen, one of the authors. The dried leaves were extracted with three times cold MeOH, and then the different partition of crude extracts was prepared with the differential proportion solvents system, including EtOAc:H_2_O, *n*-hexane: EtOAc, acetone: H_2_O, and *n*-hexane: acetone for further isolating the secondary metabolites. Seven sesquiterpenoids, (e.g., (-)-ent-6*α*-methoxyeudesm-4(15)-en-1*β*-ol, hiiranlactones A–D, (+)-villosine, hiiranepoxide), one triterpenoid (hiiranterpenone), and 22 known compounds were identified and elucidated by spectroscopic analysis and single crystal X-ray diffraction (Liou et al., 2011). An established QSAR model for drug-induced liver injury (DILI) was utilized for prediction of non/less toxic pure compounds for further functionality tests (Huang et al., 2015).

### Reagents and chemicals

All reagents were purchased from Sigma (St Louis, MO) unless otherwise stated. Fetal bovine serum (FBS) and cell culture medium RPMI 1640 were used from Hyclone (Logan, UT). Enzyme-linked immunosorbent assay (ELISA) sets for cytokine and antibody measurement were purchased from BD Biosciences (San Diego, CA). Isol-RNA lysis reagent were purchased from 5-Prime (Gaithersburg, MD). RevertAid RT kit was purchased from Thermo for Reverse transcription-polymerase chain reactions (RT-PCR).

### Animals

Male BALB/c mice (5 weeks old) were purchased from BioLasco (Ilan, Taiwan). On arrival, mice were randomly transferred to plastic cages containing aspen bedding (five mice per cage) and acclimatized for at least one week before initiating experiments. Mice were housed in a temperature (22 ± 2 °C), humidity (50 ± 20%) and light (12-hour light/dark cycle)-controlled environment. Food and water were supplied *ad libitum*.

### Animal model for antigen-specific T-cell function

**Figure 1:**
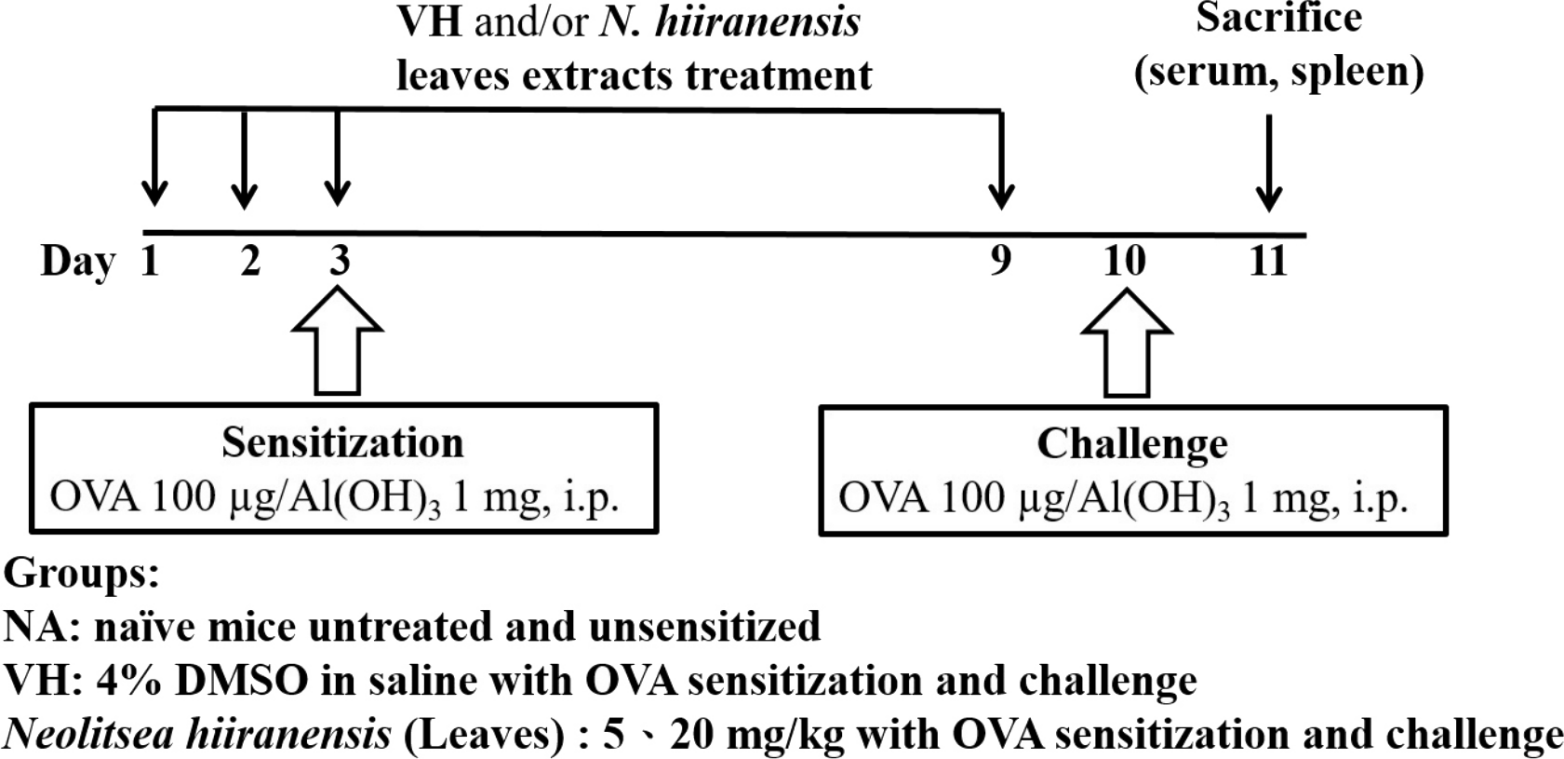
Protocol of administration of *N. hiiranensis* and ovalbumin (OVA) sensitization and challenge in BALB/c mice. Male BALB/c mice were randomly divided into the following groups: naïve (NA), vehicle (VH; Saline, and 4% of DMSO) and *N. hiiranensis*-treated (5 and 20 mg/kg) plus ovalbumin-sensitized and challenged groups. The mice were administered with VH and/or crude extracts by intraperitoneal injection for four doses. The dosing regimen for administration of *N. hiiranensis* and immunization protocol were described in the materials and methods.

The experimental protocol was approved by the Kaohsiung Medical University Institutional Animal Care and Use Committee (IACUC number 101132). Mice were administered daily by intraperitoneal injection of crude extracts (5 and 20 mg/kg) for three doses before antigen sensitization. The protocol was shown in Fig. 1. Mice were randomly divided into the following groups: naïve control (NA), vehicle (4% DMSO)-treated group (VH), and *N. hiiranensis*-treated groups (5 and 20 mg/kg in 4% DMSO). Vehicle and/or *N. hiiranensis* were administered to mice daily by intraperitoneal injection for three consecutive days (day 1–3). Except for the NA group, mice were sensitized with OVA 12 h after the third dose of VH or *N. hiiranensis* on day 3 by an intraperitoneal injection with 0.1 mL per mouse of sensitization solution containing 100 µg OVA and 1 mg alum (as adjuvant) in saline. The mice and then challenged with OVA/alum at day 9. After OVA challenge, the mice were sacrificed at day 10 and their spleens were prepared and made into single-cell suspensions. The splenocytes were re-stimulated with OVA (100 µg/mL) in culture for 72 h to induce cell proliferation and cytokine production.

### Cell proliferation assay

Splenocytes from the mice were aseptically cultured in RPMI 1640 medium supplemented with 5% heat-inactivated FBS, 100 µg/mL streptomycin, and 100 U/mL penicillin at 37 °C in 5% CO_2_. Splenocytes (7 × 10^6^ cells/mL) were seeded into 96-well plates. The cells were either left unstimulated or stimulated with OVA for 72 h. The viability of splenocytes was determined by the 3-(4, 5-dimethylthiazol-2-yl)-2, 5-diphenyl-tetrazolium bromide (methylthiazol tetrazolium) assay. A methylthiazol tetrazolium stock solution (5 mg/mL in phosphate buffered saline) was then added to each well (10 µL/well) and incubated for 4 h. The formed formazan was dissolved with a lysis buffer (10% SDS in *N*, *N*-demethylformamide) overnight in the dark. The optical density was measured at 570 nm (and at 630 nm as a background reference) using a microplate reader (Dynatech Laboratories Inc, Chantilly, VA, USA).

### Enzyme-linked immunosorbent assay (ELISA) for serum antibodies

ELISA plates were coated with 0.05% OVA in coating buffer (0.1 M NaHCO_3_) and blocked with 1% bovine serum albumin in phosphate-buffered saline containing 0.05% Tween 20 (PBST). After washing with PBST, the serum samples were added into wells (50 µL/well) and incubated for 1 h. After another washing, horseradish peroxidase-conjugated anti-mouse IgG_1_, IgG_2a_ or IgM was added (50 µL/well) and incubated for 1 h. Finally, wells were washed and a tetramethylbenzidine solution (50 µL/well) was added for colorimetric detection of bound peroxidase conjugate. The reaction was terminated by adding 150 µL of 3 N H_2_SO_4_ per well. The optical density (OD) was measured at 450 nm using a microplate reader (Dynatech Laboratories, Chantilly, VA, USA). Total IgE was measured according to manufacture’s instruction (BD Pharmingen).

### Cytokine measurement by ELISA

To examine the effects of *N. hiiranensis* on specific subsets of T cells, splenocytes (7 × 10^6^ cells/mL) were cultured in 48-well plates (300 µL/well) followed by OVA re-stimulated (100 µg/mL) for 72 h. The supernatants were harvested and quantified for IL-2, IL-4, IL-12p70, IL-10, IL-13 and IFN-*γ* by ELISA kits according to manufacture’s instruction (BD Pharmingen).

### *In silico* prediction of hepatotoxicity and genotoxicity

Quantitative structure-activity relationship (QSAR) models are useful tools for *in silico* estimating toxicity properties of chemicals according to toxicity-related descriptors of physicochemical properties and fingerprints (Perkins et al., 2003). QSAR models have been extensively applied to prioritize chemicals for potential toxicity (Cronin et al., 2003; Gramatica, Cassani & Sangion, 2016). In this study, toxicity properties of tested compounds were predicted by both the admetSAR server (Cheng et al., 2012) and our hepatotoxicity prediction model (Huang et al., 2015). The genotoxicity, carcinogenicity and acute oral toxicity of tested compounds are predicted by admetSAR with probabilities representing the confidence of prediction. The hepatotoxicity model is a special QSAR model utilizing toxicity information in human that no cross-species extrapolation is required. Similar to admetSAR, a confidence score is given by the hepatotoxicity prediction model. Generally, a score/probability close to 1 indicates a higher probability that a toxicity is associated with a given chemical. In contrast, a score closed to 0 indicates that a toxicity is unlikely associated with a given chemical.

### RNA isolation and real-time reverse transcription-polymerase chain reactions (RT-PCR)

Total RNA from whole splenocytes (stimulated with OVA for 48 h) was isolated using an isol-RNA lysis reagent (5-Prime). The RNA samples (5 µg) were then treated with an RQ1 RNase-free DNase kit (Promega, Southampton, UK) according to the manufacturer’s instructions to remove contaminated DNA and the quality of total RNA was confirmed by agarose gel electrophoresis. The RNA concentration of each sample were quantified using determination of optical density at 260 nm (OD260) by a microplate reader (Thermo varioskan flash; Thermo Fisher Scientific, Waltham, MA, USA). One µg of total RNA of each sample was reverse-transcribed by RevertAid RT Kit (Thermo) into cDNA products using oligo (dT) as primer. Real-time PCR was performed in a 96-well optic tray by an ABI PRISM^®^ 7900HT Sequence Detection System (Applied Biosystems, UK). During real-time RT-PCR process, we used Luminaris Color HiGreen High ROX qPCR Master Mix (Thermo Fisher Scientific, Waltham, MA, USA) which provides a highly specific and sensitive method to quantify mRNA expression. The HPRT gene was used as an endogenous control to normalize the expression of target genes. The primers are: 5^′^-GCCAGGGAACCGCTTATATG-3^′^ and 5^′^-GACGATCATCTGGGTCACATTCT-3^′^ for T-bet, 5^′^-TACCCTCCGGCTT- CATCCT-3^′^ and 5^′^-TGCACCTGATACTTGAGGCAC-3^′^ for GATA-3, 5^′^-GCC- AAGTTTGAGGTCAACAAC-3^′^ and 5^′^-CCGAATCAGCAGCGACTC-3^′^ for IFN-*γ*, 5^′^-CCATATCCACGGATGCGACA-3^′^ and 5^′^-AAGCCCGAAAGAGTCTCTGC-3^′^ for IL-4, 5^′^-TCAGTCAACGGGGGACATAAA-3^′^ and 5^′^-GGGGCTGTA- CTGCTTAACCAG-3^′^ for HPRT (Shi et al., 2010), and 5^′^-CCTCAATGGTATAGCAGAAC and 5^′^ -TAGCCTTGG AATCCTTGG for IL-12R*β*2 (Chognard et al., 2014).

### *In vitro* screening of cytokine production by antigen-specific T cells

OVA-primed splenocytes were generated according to previous protocol. Briefly, 6–8 week mice were sensitized by intraperitoneal injection of OVA (10 mg OVA absorbed to 100 mg alum as adjuvant) twice on day 1 and 14. On day 15, the mice were sacrificed and their spleens were harvested and made into single-cell suspensions. The OVA-primed splenocytes (7 × 10^6^ cells/mL) were either left untreated (control), 0.05% DMSO (VH) and/or secondary metabolites (1–10 µM) followed by re-stimulation with OVA (100 µg/mL) for 72 h. The cell proliferation activity, cytokine productions, and mRNA expression of target genes were measured as described above.

### Flow cytometry analysis of intracellular cytokine and transcription factor staining in CD4^+^ cells

OVA-primed splenocytes were cultured in a 12-well plate and stimulated with OVA and *β*-caryophyllene oxide for 36 h. For analysis of intracellular cytokine production, the cells then treated with GolgiStop (0.6 µL/mL; BD Biosciences) for 10 h prior to being harvested for antibody staining. The cells were next stained with FITC-conjugated anti-mouse CD4 mAb (clone GK1.5; Biolegend, CA, USA) for 30 min on ice. The splenocytes then were fixed and permeabilized using Fixation and Perm/Wash buffers (BD Biosciences) before staining for intracellular IFN-*γ* by PE-conjugated anti-mouse IFN-*γ* mAb (clone XMG1.2; Biolegend) for 30 min on ice. Ten thousand CD4^+^ cells were acquired on a BD LSR II flow cytometer (BD Biosciences). The mean fluorescence intensity (MFI) of IFN-*γ* in total CD4^+^ cells was quantified by gating CD4^+^ cells and then analyzed using FlowJo software (Treestar, Inc., CA). For detection of T-bet and GATA-3, splenocytes treated with *β*-caryophyllene oxide for 36 h and then harvested for anti-CD4 mAb staining. Next the cells were fixed and permeabilized using True-Nuclear Transcription Factor Buffer Set (Biolegend) according to the manufacturer’s instructions. PerCP-Cy5.5-conjugated anti-mouse T-bet mAb (clone 4B10; Biolegend) and PerCP-Cy5.5 conjugated anti-mouse GATA-3 mAb (clone 16E10A23; Biolegend) were applied to detect protein level of T-bet and GATA-3 in CD4^+^ cells. Ten thousand CD4^+^ cells were acquired on a BD LSR II flow cytometer (BD Biosciences). The mean fluorescence intensity (MFI) of T-bet or GATA-3 in total CD4^+^ cells was quantified by gating CD4^+^ cells and then analyzed using FlowJo software (Treestar, Inc., Ashland, OR, USA).

### Statistical analysis

Homogeneous data were evaluated by a parametric analysis of variance (ANOVA) with Dunnett’s test to assess the statistical differences between the treatment groups and the VH control group by software Prism 5.0 (GraphPad Software Inc., San Diego, CA, USA). *P* < 0.05 was defined as statistical significance. The mean ± standard error was presented for individual experiments.

## Results

### Crude extracts of leaves of *N. hiiranensis* did not affect body weight, spleen index, and cellularity *in vivo*

**Figure 2:**
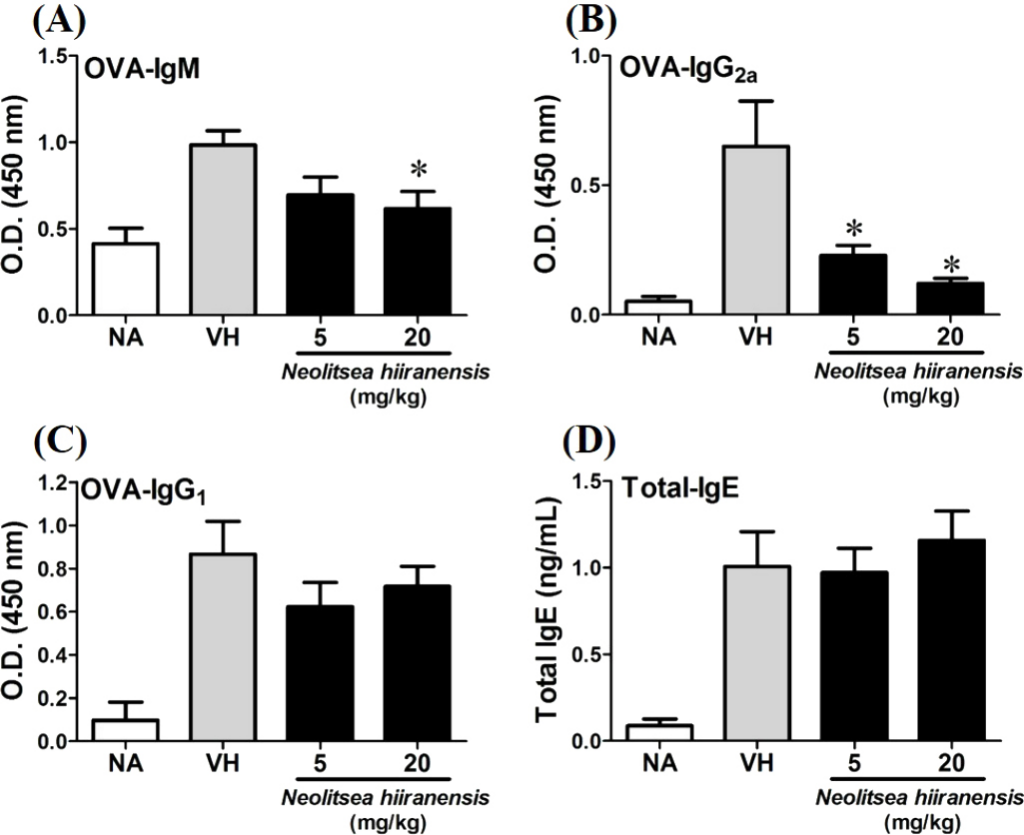
*N. hiiranensis* attenuated OVA-specific IgM and IgG_2a_ production. (A–D) The serum levels of OVA-specific IgM, IgG_2a_ IgG_1_, and total-IgE were determined by ELISA. Data are expressed as the mean ± standard error of eight samples per group. Results are pooled from three independent experiments. *p <0.05 compared to the vehicle-treated group.

To investigate the potential of *N. hiiranensis* for *in vivo* use, we first investigated the direct immunotoxicity of *N. hiiranensis in vivo*. As shown in Table 1, intraperitoneal injection of *N. hiiranensis* extracts (5 and 20 mg/kg) didn’t affect the body weight, the spleen index, and the population of CD4^+^, CD8^+^, B220^+^, and CD11b^+^ in spleens of the mice received treatment. We then examined the effect of the *N. hiiranensis* extracts on T-cell mediated humoral and cell-mediated immune responses *in vivo*. The *N. hiiranensis* extracts decreased the serum level of OVA-specific IgM and IgG_2a_. The serum level of OVA-specific IgG_2a_ was dose-dependently suppressed by *N. hiiranensis* extracts. At the dose of 20 mg/kg, the level of OVA-specific IgM was significantly decreased. In contrast, *N. hiiranensis* didn’t affect OVA-specific IgG_1_ and total-IgE production. The result indicated that repeating administration of *N. hiiranensis* significantly suppressed Th1-associated antibody production (Fig. 2)

**Table 1:** No effect of *N. hiiranensis* leaves extracts on the body weight, spleen index and cellularity in BALB/c mice.

	NA^a^	VH	Leaves extracts of *N. hiiranensis*	
			5 mg/kg	20 mg/kg
Body weight (g)				
Day1	22.0 ± 0.3	22.7 ± 0.5	22.0 ± 0.3	22.5 ± 0.4
Day9	22.6 ± 0.3	23.7 ± 0.5	23.8 ± 0.2	23.7 ± 0.4
Spleen weight (mg)	91.4 ± 3.1	103.5 ± 4.2	97.1 ± 4.5	91.8 ± 3.4
Spleen index^b^	4.1 ± 0.2	4.4 ± 0.2	4.2 ± 0.2	3.9 ± 0.2
Spleen cellularity (%)^c^				
CD4^+^	21.1 ± 0.3	22.6 ± 0.3	23.2 ± 0.7	22.6 ± 0.7
CD8^+^	10.2 ± 0.3	11.3 ± 0.2	12.3 ± 0.5	11.5 ± 0.2
B220^+^	45.9 ± 1.0	45.4 ± 1.0	46.2 ± 1.1	46.1 ± 0.2
CD11b^+^	2.5 ± 0.3	2.5 ± 0.3	2.5 ± 0.1	2.5 ± 0.0

**Notes.**

a NA, untreated; VH, vehicle-treated and OVA-sensitized and challenged and leaves: *N. hiiranensis* -treated and OVA-sensitized and challenged.

b Spleen index was calculated as the spleen weight (mg) per body weight (g). Data are expressed as mean ± SE of eight (control groups) and eleven (treatment groups) mice from three independent experiments.

c The percentage of CD4^+^, CD8^+^, B220^+^, and CD11b^+^cells in spleen was determined by flow cytometry. Data are expressed as mean ± SE of four samples pooled from three independent experiments

**Figure 3:**
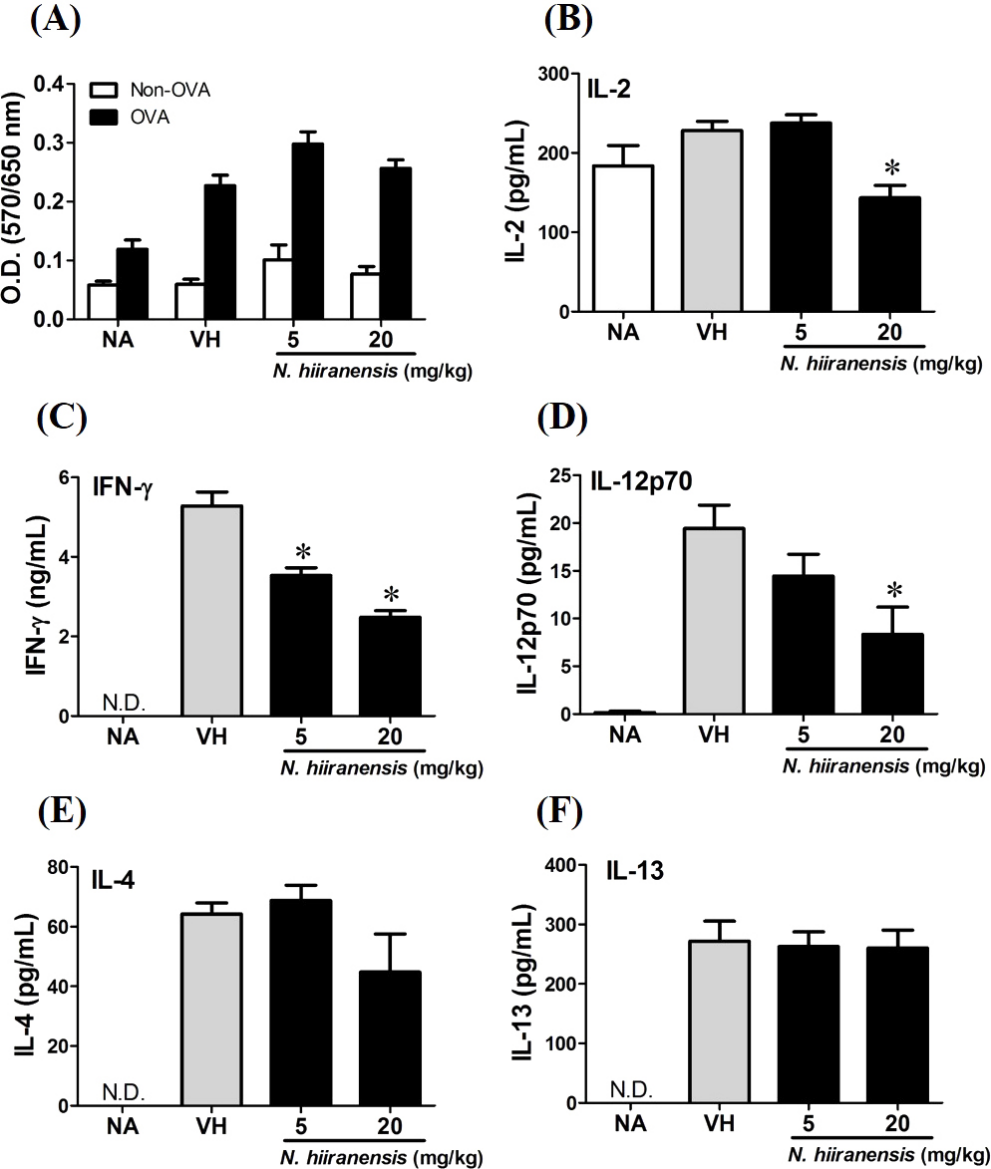
Suppression of IL-2, IFN-*γ*, and IL-12 production by leave extracts of *N. hiiranensis in vivo*. Splenocytes with same cell concentration were prepared from each group of mice and cultured in the presence of ovalbumin (100 µg/mL) for 72 h. The MTT assay was applied to determine the proliferation activity after re-stimulation of OVA *ex vivo* (A). The supernatants were collected for measuring the concentration of IL-2, IFN-*γ*, IL-12, IL-4, and IL-13 by ELISA (B–F). N.D. indicated no detectable level of cytokines in the supernatant. Data were expressed as the mean ± SE of quadruplicate cultures. Results were representative of three independent experiments. *p < 0.05 was significant compared to the VH group.

### Crude extracts of leaves of *N. hiiranensis* attenuated antigen-specific IL-2, IL-12, and IFN-*γ* cytokine production *in vivo*

We then proceed to examine the effects of the crude extracts on the functionality of antigen-specific T cells by measuring the production of IL-2 (T-cell growth factor for clonal expansion), IFN-*γ* and IL-4 (the signature Th1 and Th2 differential cytokines), IL-12 (induction of IFN-*γ* production and Th1 differentiation), and IL-13 (Th2 cytokine closely related IL-4 to induce allergic Th2 responses). To check whether or not the induction of OVA was successful, the splenocytes were isolated and divided into non-stimulated (Non-OVA) and OVA 100 µg/mL re-stimulated (OVA) groups and culture for 72 h to induce antigenspecific cytokine production. The result showed induced cell proliferation in OVA-restimulated groups compared to their corresponding non-stimulated groups (Fig. 3A). In addition, the leaves extractions of *N*. *hiiranensis* did not affect the proliferation of OVA-specific splenocytes. Despite not affecting cell proliferation, the extracts significantly decrease the production of antigen-specific IL-2 and IL-12 in the *N*. *hiiranensis* 20 mg/kg group (Figs. 3B and 3D). The production of IFN-*γ* by the cells was also attenuated by the treatment. IFN-*γ* was significantly lowered at both 5 mg/kg and 20 mg/kg treatment groups (*P* < 0.05 for each group compared to the vehicle control group; Fig. 3C). By contrast, IL-4 and IL-13 were not affected (Figs. 3E and 3F). The results indicated that the extracts of the leaves of *N. hiiranensis* have differential effects on Th1 responses.

### *In silico* selection of potential secondary metabolites of *N. hiiranensis* without undesired toxicity

**Figure 4:**
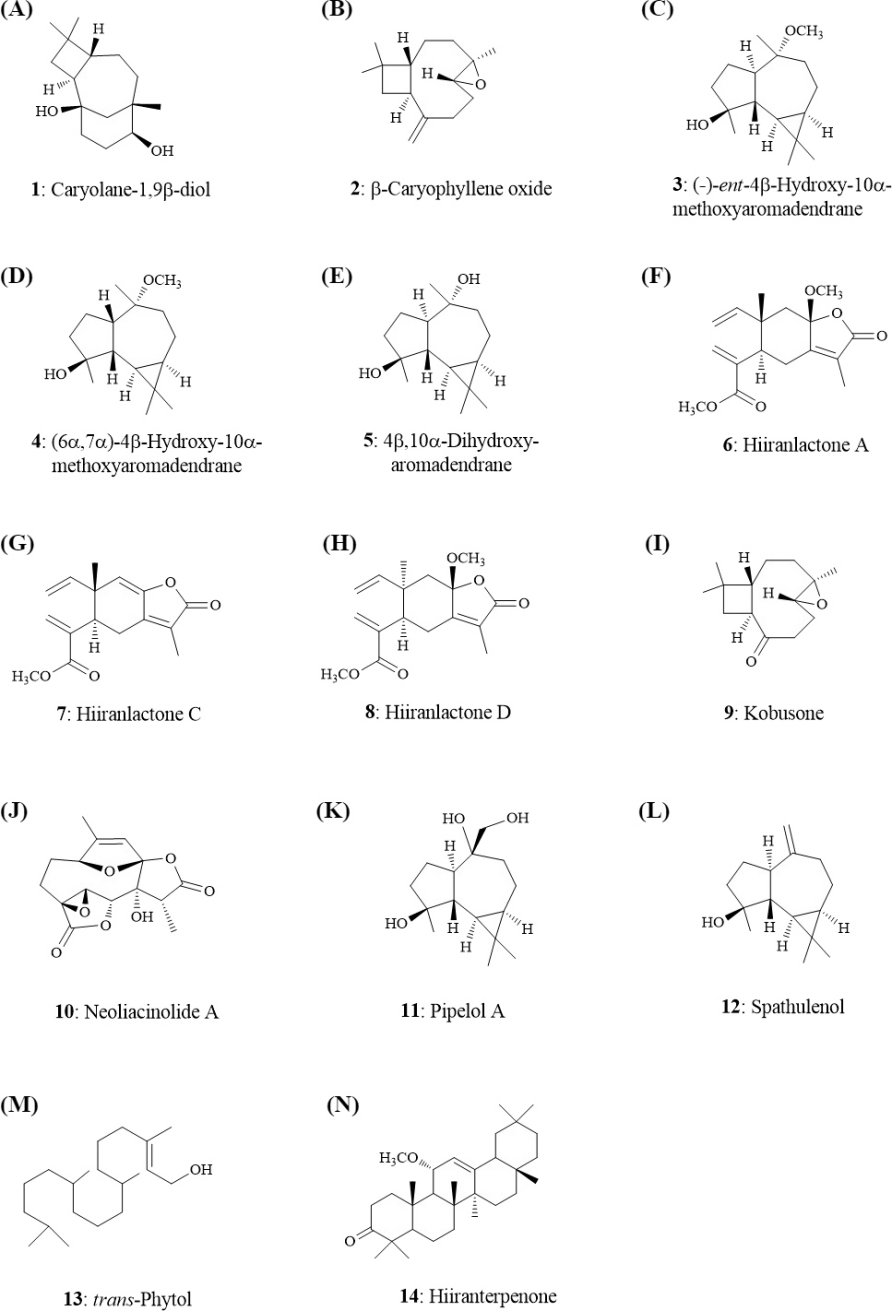
Structures of the secondary metabolites (1–14) from the leaves of *N. hiiranensis*.

Quantitative structure–activity relationship (QSAR) models of admetSAR (Cheng et al., 2012) and our hepatotoxicity prediction model (Huang et al., 2015) were applied to predict genotoxicity, carcinogenicity, and hepatotoxicity of 14 secondary metabolites from *N*. *hiiranensis* (Fig. 4). We firstly converted our tested compounds into SMILES (Simplified Molecular Input Line Entry System) representations, a line notation for representing the structure of molecules and reactions. The SMILES is subsequently submitted to admetSAR and our hepatotoxicity model to predict their genotoxic, carcinogenic, and hepatotoxic potentials. Table 2 showed the predicted toxicity of 14 compounds. The probability of genotoxicity (Ames Test), carcinogenicity, and hepatotoxicity among the 14 selected compounds were 0.05–0.49, 0.07–0.24, and 0.45–0.60, respectively, indicating that these selected compounds were classified as AMES-negative, non-carcinogenic and less hepatotoxic compounds. Moreover, the estimated acute oral toxicity of these compounds was predicted as class III (US EPA category system; LD_50_ value is between 500–5,000 mg/kg) with the probability of 0.45–0.83.

### Secondary metabolites of *N. hiiranensis* attenuated IFN-*γ* production *in vitro*

**Figure 5:**
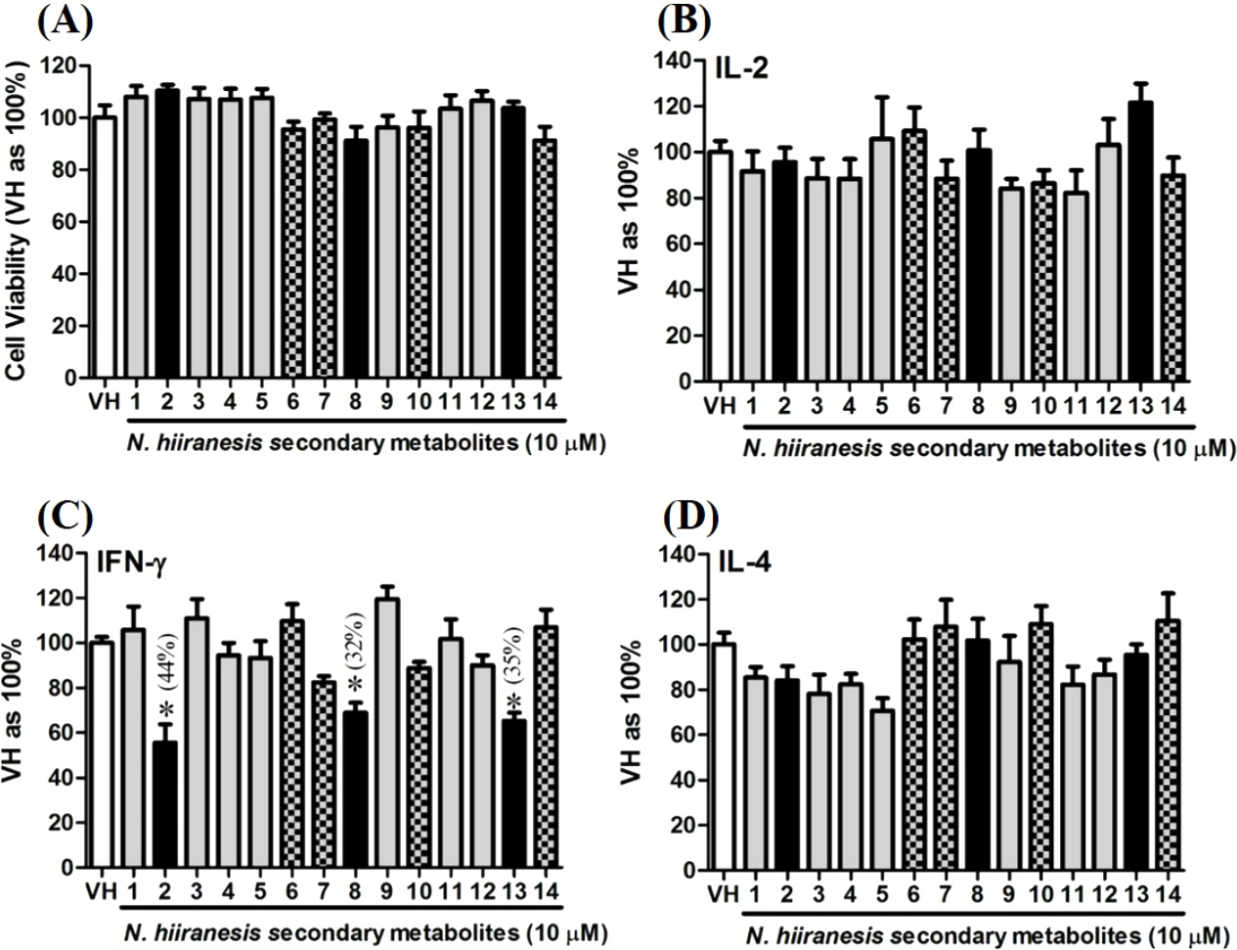
The effects of secondary metabolites of leaves of *N. hiiranensis* on antigen-induced production of cytokines and the metabolic activity in OVA-primed splenocytes. OVA-primed splenocytes (7 × 10^6^cells/mL) isolated from OVA-sensitized BALB/c mice were pretreated with secondary metabolites (10 µM) and/or VH (0.05% DMSO) for 30 min followed by re-stimulated OVA (100 µg/mL). After 72 h of culture, (A) the cell proliferation activity was determined using an MTT assay, the level of (B) IL-2, (C) IFN-*γ*, and (D) IL-4 in the supernatants was quantified by ELISA. The data were expressed as the mean ± SEM of quadruplicate cultured. Results were pooled from two or three independent experiments. *p < 0.05 was significant compared to the VH group.

OVA-prime splenocytes were generated for screening the immunomodulatory effects of the compounds from *N*. *hiiranensis*. The OVA-primed splenocytes were re-stimulated with OVA (100 µg/mL) in the presence of vehicle and/or 10 µM of selected compounds for 72 h *in vitro*. The selected pure compounds didn’t affect the proliferation activity nor induce the direct cytotoxicity (Fig. 5A). IL-2 and IL-4 were not significantly affected by the 14 selected compounds compared to VH. Interestingly, the antigen specific IFN-*γ* cytokine production were significantly suppressed by *β*-caryophyllene oxide (**2**), hiiranlactone D (**8**), and *trans*-phytol (**13**) with the inhibition rate of 44%, 32%, and 35%, respectively, comparing to VH (referred as 100%) (*P* < 0.05). Spathulenol (**12**) slightly inhibited IFN-*γ* and IL-4 productions without statistical significance (Fig. 5).

**Table 2:** The toxicity profile of 14 selected secondary metabolites from *N. hiiranensis*. In order to select less toxic compounds for further study, *in silico* QSAR models were applied to filter out the compounds with potential toxicity concerns. A probability ≦0.5 indicates no toxicity concern. A probability ≦0.6 indicates a less hepatotoxicity concern.

Secondary metabolites	Formula	Classification	**Predicted probability**		
			AMES Toxicity	Carcinogens	Hepatotoxicity
Caryolane-1,9*β*-diol	C_15_H_26_O_2_	Sesquiterpenoids	0.41	0.13	0.45
*β* −Caryophyllene oxide	C_15_H_24_O	Sesquiterpenoids	0.05	0.23	0.50
(-)-*ent*-4*β*-Hydroxy-10*α*-methoxyaromadendrane	C_16_H_28_O_2_	Sesquiterpenoids	0.21	0.15	0.49
(6*α*,7*α*)-4*β*-Hydroxy-10*α*-methoxyaromadendrane	C_16_H_28_O_2_	Sesquiterpenoids	0.21	0.15	0.46
4*β*,10*α*-Dihydroxyaromadendrane	C_15_H_26_O_2_	Sesquiterpenoids	0.21	0.15	0.50
Hiiranlactone A	C_17_H_22_O_5_	Sesquiterpenoids	0.11	0.10	0.58
Hiiranlactone C	C_16_H_18_O_4_	Sesquiterpenoids	0.11	0.10	0.60
Hiiranlactone D	C_17_H_22_O_5_	Sesquiterpenoids	0.11	0.10	0.58
Kobusone	C_14_H_22_O_2_	Sesquiterpenoids	0.10	0.17	0.53
Neoliacinolide A	C_15_H_16_O_7_	Sesquiterpenoids	0.49	0.07	0.60
Pipelol A	C_15_H_26_O_3_	Sesquiterpenoids	0.21	0.15	0.51
Spathulenol	C_15_H_24_O	Sesquiterpenoids	0.24	0.11	0.46
*trans*-Phytol	C_20_H_40_O	Diterpenoids	0.16	0.24	0.52
Hiiranterpenone	C_31_H_50_O_4_	Triterpenoids	0.06	0.10	0.56

**Figure 6:**
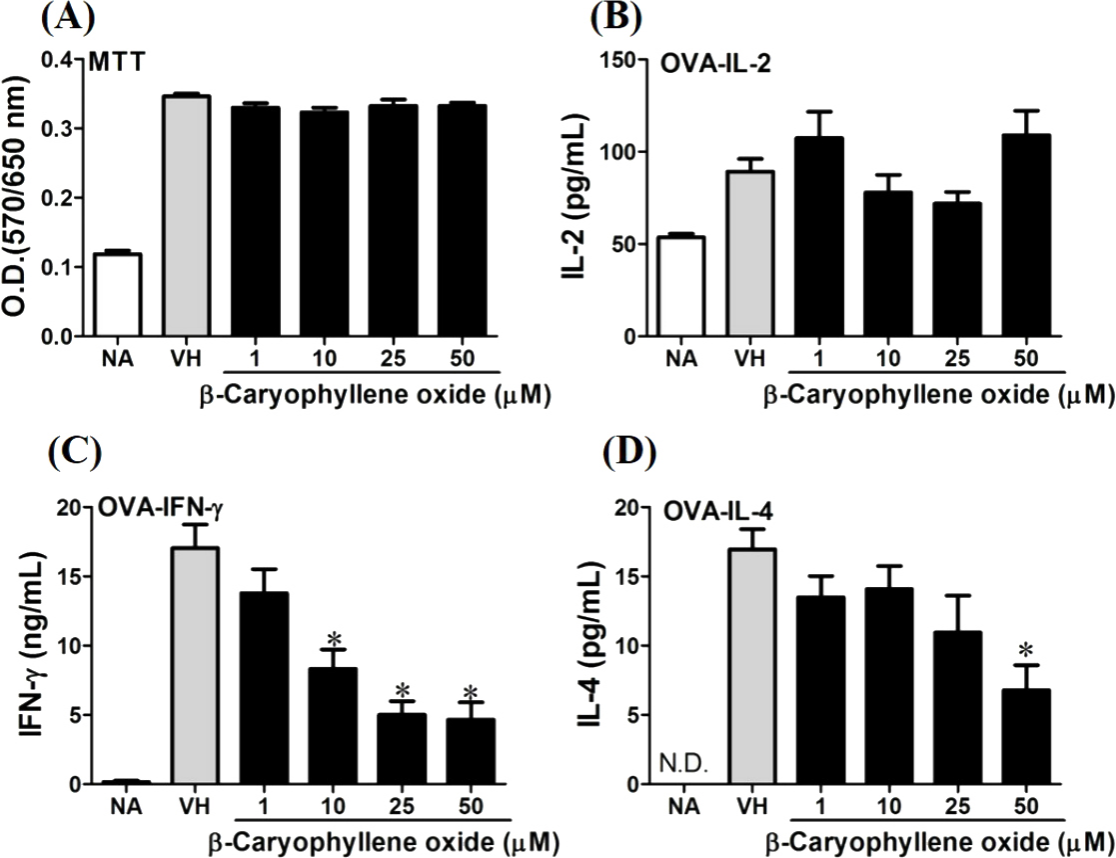
Antigen-specific IFN-*γ* was suppressed by *β*-caryophyllene oxide *in vitro*. OVA-primed splenocytes (7 × 10^6^cells/mL) were either left untreated (NA) or re-stimulated with OVA (100 µg/mL) in the absence or the presence of *β*-caryophyllene oxide (1–50 µM) for 72 h. (A) The cell proliferation activity of viable cells was determined using the MTT assay. The levels of (B) IL-2, (C) IFN-*γ*, and (D) IL-4 in the supernatants were quantified by ELISA. Data were expressed as the mean ± SE of quadruplicate cultures. Results were pooled from two independent experiments. *p < 0.05 was significant compared to the VH group.

**Figure 7:**
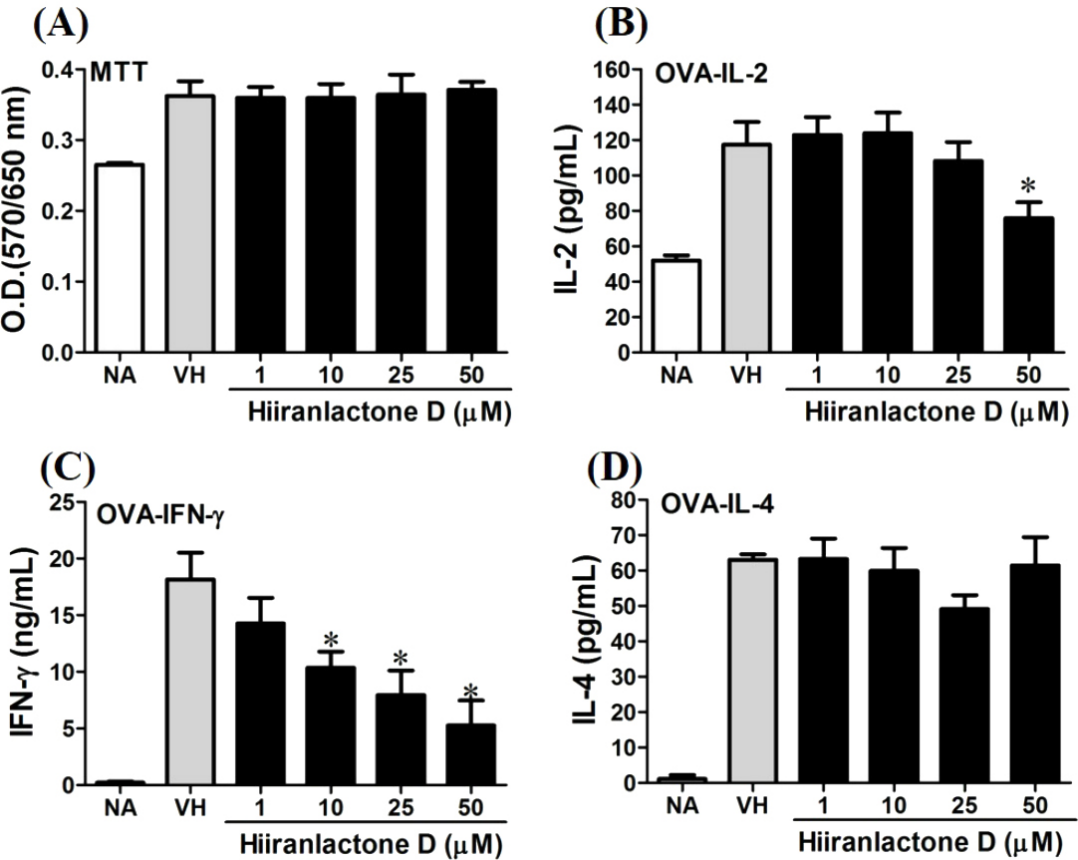
Attenuation of antigen-specific IFN-*γ* production by hiiranlactone D. OVA-primed splenocytes were treated with various concentration of hiiranlactone D (1–50 µM) in the presence of ovalbumin (100 µg/mL) for 72 h. (A) The cell proliferation activity of treated cells was determined using the MTT assay. The concentration of (B) IL-2, (C) IFN-*γ*, and (D) IL-4 in the supernatants was measured by ELISA. Data were expressed as the mean ± SE of quadruplicate cultures. Results were pooled from two independent experiments. *p < 0.05 was significant compared to the VH group.

**Figure 8:**
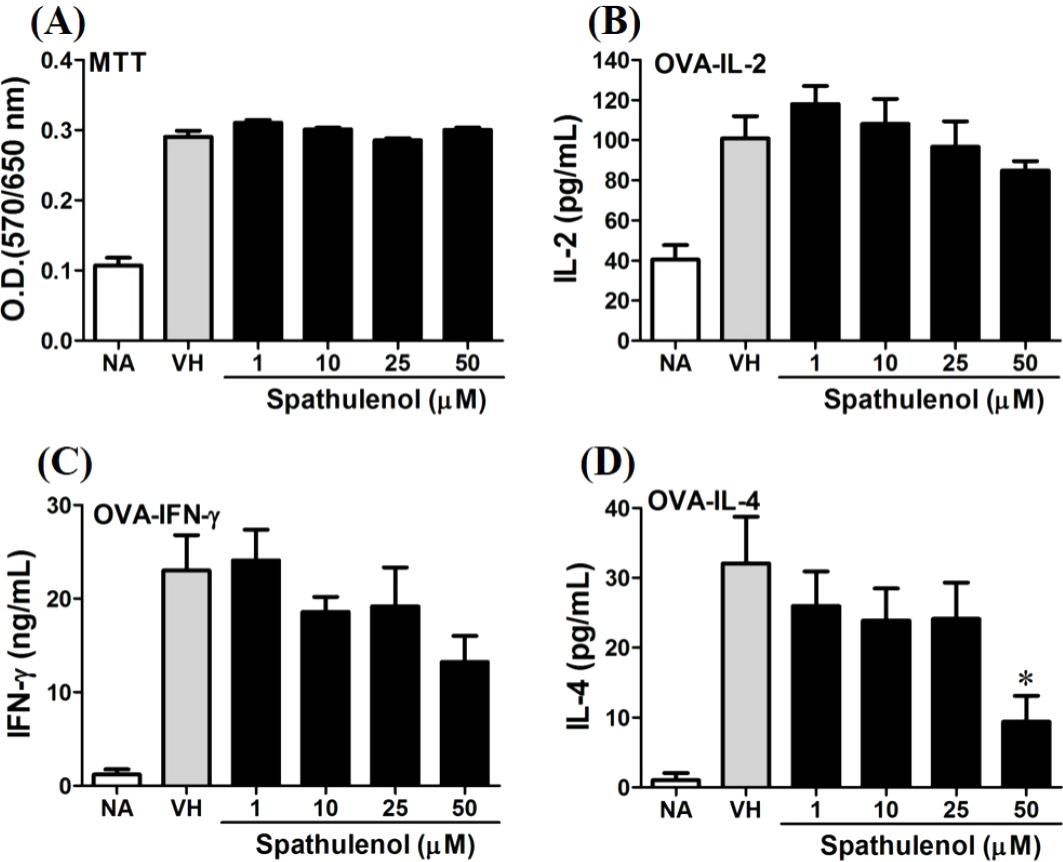
Spathulenol slightly inhibited IL-4 production at high concentration. OVA-primed splenocytes were treated with various concentration of spathulenol (1–50 µM) in the presence of ovalbumin (100 µg/mL) for 72 h. (A) The cell proliferation activity was determined using the MTT assay. The concentration of (B) IL-2, (C) IFN-*γ*, and (D) IL-4 in the supernatants was measured by ELISA. Data were expressed as the mean ± SE of quadruplicate cultures. Results were pooled from two independent experiments. *p < 0.05 was significant compared to the VH group.

**Figure 9:**
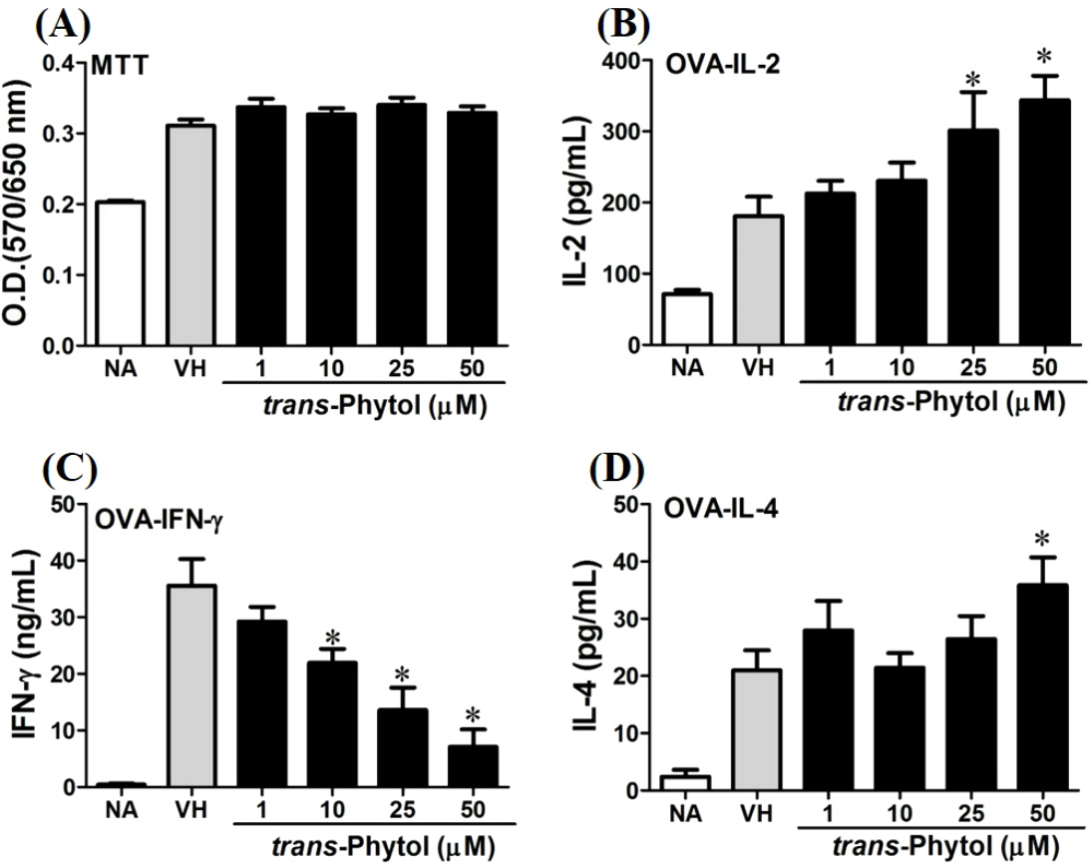
*trans*-Phytol differentially modulated Th1/Th2 cytokine production *in vitro*. OVA-primed splenocytes were treated with various concentration of *trans-*phytol (1–50 µM) in the presence of ovalbumin (100 µg/mL) for 72 h. (A) The cell proliferation activity was determined using the MTT assay. The concentration of (B) IL-2, (C) IFN-*γ*, and (D) IL-4 in the supernatants was measured by ELISA. Data were expressed as the mean ± SE of quadruplicate cultures. Results were pooled from two independent experiments. *p < 0.05 was significant compared to the VH group.

We further investigated the concentration-dependent effects of these compounds (**2**, **8**, **12**, and **13**) on cytokine production. The effects of *β*-caryophyllene oxide (**2**), hiiranlactone D (**8**), spathulenol (**12**), and trans-phytol (**13**) on the cell viability and Th1/Th2 cytokine secretions are shown in Figs. 6–9. *β*-caryophyllene oxide (1–50 µM) didn’t alter the proliferation activity as well as IL-2 cytokine production of OVA-specific cells. By contrast, *β*-caryophyllene oxide inhibited IFN-*γ* production in a dependent manner and inhibited IL-4 production with an approximately 40% of inhibition rate at the concentrations higher than 25 µM (Fig. 6). Hiiranlactone D (**8**) didn’t affect cell viability at the concentration of 50 µM. Hiiranlactone D attenuated IL-2 production of OVA-specific cells at 50 µM and inhibited IFN-*γ* production in a concentration dependent manner starting from concentrations above 10 µM (*P* < 0.05). No effect on IL-4 was observed for hiiranlactone D (Fig. 7). Spathulenol also didn’t affect cell viability at the concentration of 50 µM. Spathulenol inhibited IL-4 production at 50 µM, while neither IL-2 nor IFN-*γ* were significantly affected (Fig. 8). *trans*-Phytol didn’t affect cell viability at the concentration of 50 µM. Interestingly, *trans*-phytol enhanced antigen-specific IL-2, and IL-4 production in an concentration-dependent manner with significant inhibitions started from 25 and 50 µM for IL-2 and IL-4, respectively. *trans*-Phytol significantly inhibited IFN-*γ* production in a concentration-dependent manner at the concentrations between 10 and 50 µM (Fig. 9). The results demonstrated a differential immunomodulatory effects of *trans*-phytol on the Th1/Th2 cytokine expression in antigen-specific T cells.

**Figure 10:**
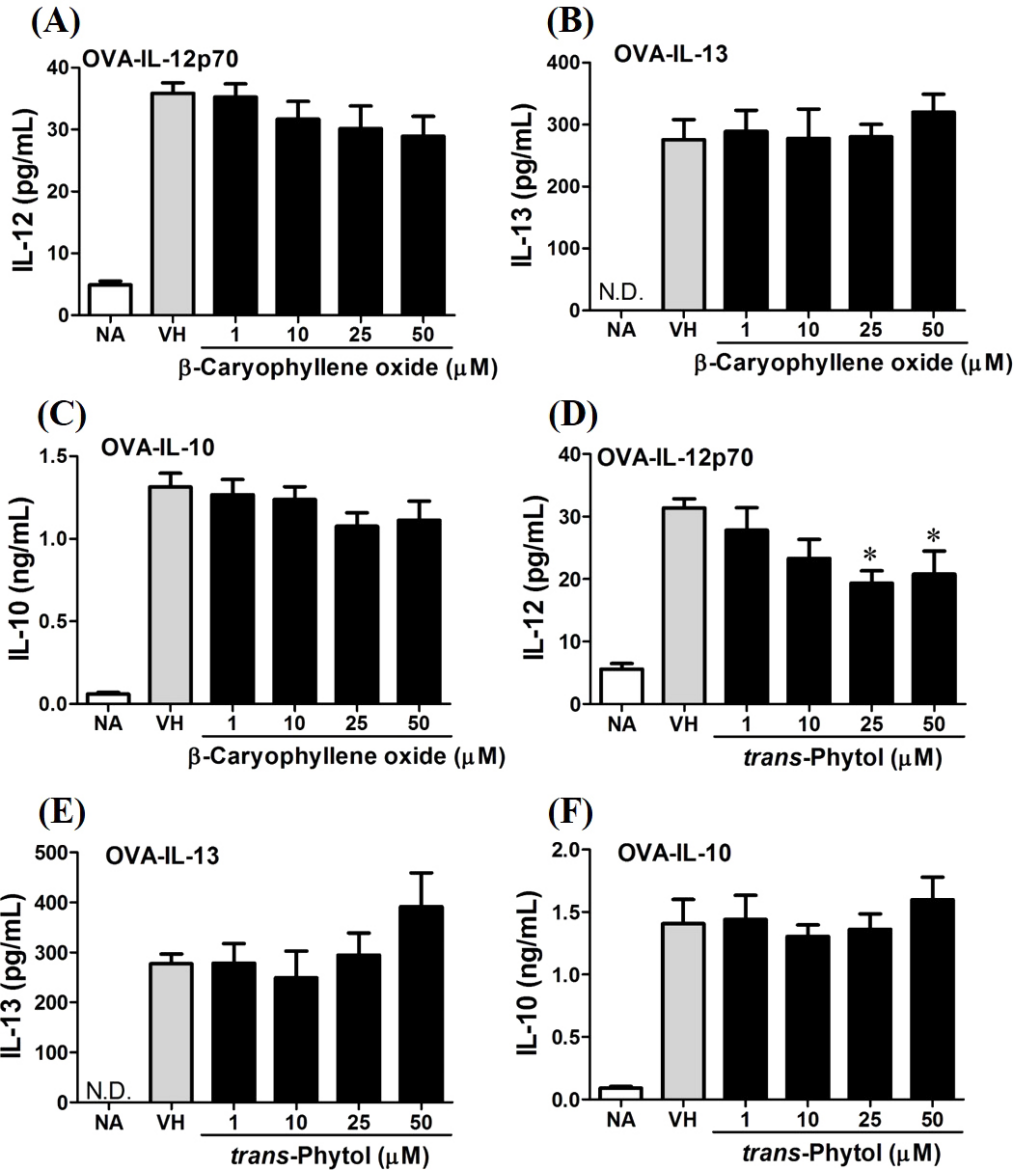
Differential effects of *β*-caryophyllene oxide and *trans*-phytol on Th1/Th2 cytokine production *in vitro*. OVA-primed splenocytes were treated with various concentration of *β*-caryophyllene oxide and *trans*-phytol (1–50 µM) in the presence of ovalbumin (100 µg/mL) for 72 h. The level of (A) IL-12, (B) IL-10, and (C) IL-13 in the supernatants of *β*-caryophyllene oxide-treated cells and (D) IL-12, (E) IL-10, and (F) IL-13 in the supernatants of *trans-*phytol-treated cells were quantified by ELISA. Data were expressed as the mean ± SE of quadruplicate cultures. Results were pooled from two independent experiments. *p < 0.05 was significant compared to the VH group.

In the present data, *β*-caryophyllene oxide and trans-phytol are the most effective secondary metabolites from *N. hiiranensis* to suppress IFN-*γ* production. We next determined the effects of *β*-caryophyllene oxide and *trans*-phytol on the production of other Th1 and Th2 cytokines, including IL-12, IL-13, and IL-10. In Fig. 10, *trans*-phytol significantly decreased IL-12 production, while both IL-13 and IL-10 were not altered (Figs. 10E and 10F). Interestingly, although *β*-caryophyllene oxide significantly suppressed IFN-*γ* production (Fig. 6C), the IL-12 production was not significantly altered by *β*-caryophyllene oxide (Fig. 10A). In order to further confirm *β*-caryophyllene oxide directly suppressed IFN-*γ* production by CD4^+^ cells, the intracellular cytokine staining approach was applied. In the supplemental data (Fig. S1), *β*-caryophyllene oxide significantly suppressed the cellular level of IFN-*γ* in the CD4^+^ cells.

### *β*-caryophyllene oxide differentially modulated the development of Th1 and Th2 cells at transcription level

**Figure 11:**
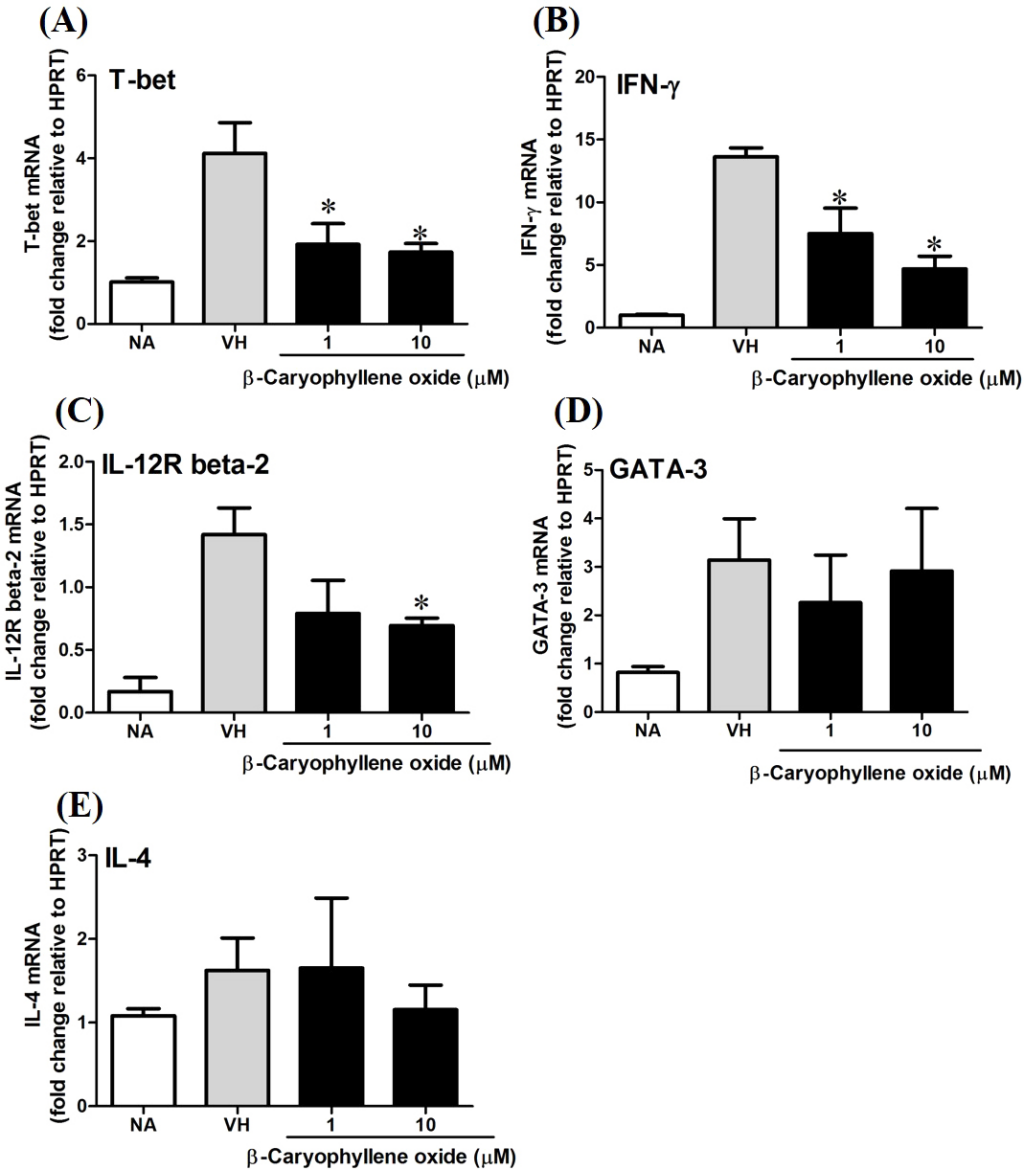
The effect of *β*-caryophyllene oxide on mRNA expression in OVA-primed splenocytes. The total RNA of splenocytes was extracted and the mRNA expression of (A) T-bet, (B) IFN-*γ*, (C) IL-12R beta-2, (D) GATA-3, and (E) IL-4 was measured by real-time RT-PCR. The expression level of HPRT was used as the control for semi-quantification. Results were expressed as the mean ± SE of pooled data from four independent experiments. *p < 0.05 was significant compared to the VH group.

T-bet is a Th1-specific T-box transcription factor which controls the expression of Th1 cytokines and directs Th1 lineage commitment, while GATA-binding protein 3 (GATA-3) is a Th2-specific transcription factor which augments Th2-specific cytokines and Th2 differentiation to suppress Th1 immune responses. These transcription factors play crucial roles to regulate the homeostasis of Th cells (Agnello et al., 2003; Koch et al., 2009; Laurence et al., 2007). To understand the effect of *N. hiiranensis* at transcription level of T-cell function, RT-PCR was performed. As *β*-caryophyllene oxide was the most effective compound selected from *N. hiiranensis* which dramatically inhibited IFN-*γ* production. We next determined how *β*-caryophyllene oxide regulates Th1/Th2 associated gene expression. IFN-*γ*, IL-4, and Th1/Th2 differential transcription factors, T-bet and GATA-3 were determined. The mRNA expression of T-bet was significantly down-regulated by approximately 2-3-fold at 1 and 10 µM (Fig. 11A), whereas GATA-3 wasn’t significantly altered after treatment of *β*-caryophyllene oxide (Fig. 11D). IFN-*γ* was also down-regulated by approximately 1.5-2-fold (Fig. 11B); however, IL-4 was not changed (Fig. 11E). As T-bet response to IFN-*γ* may lead to the up-regulation of IL-12R*β*2 expression on Th1 cell surface for Th1 cell responsiveness to IL-12 stimulation (Hamza, Barnett & Li, 2010), we next determined whether *β*-caryophyllene oxide attenuated the expression of IL-12R*β*2. In consist with IFN-*γ* expression, the IL-12R*β*2 expression was down-regulated by approximately 1.5-2-fold (Fig. 11C). These results showed that the differentiation and functionality of Th1 cells were more sensitive to be attenuated by *β*-caryophyllene oxide.

**Figure 12:**
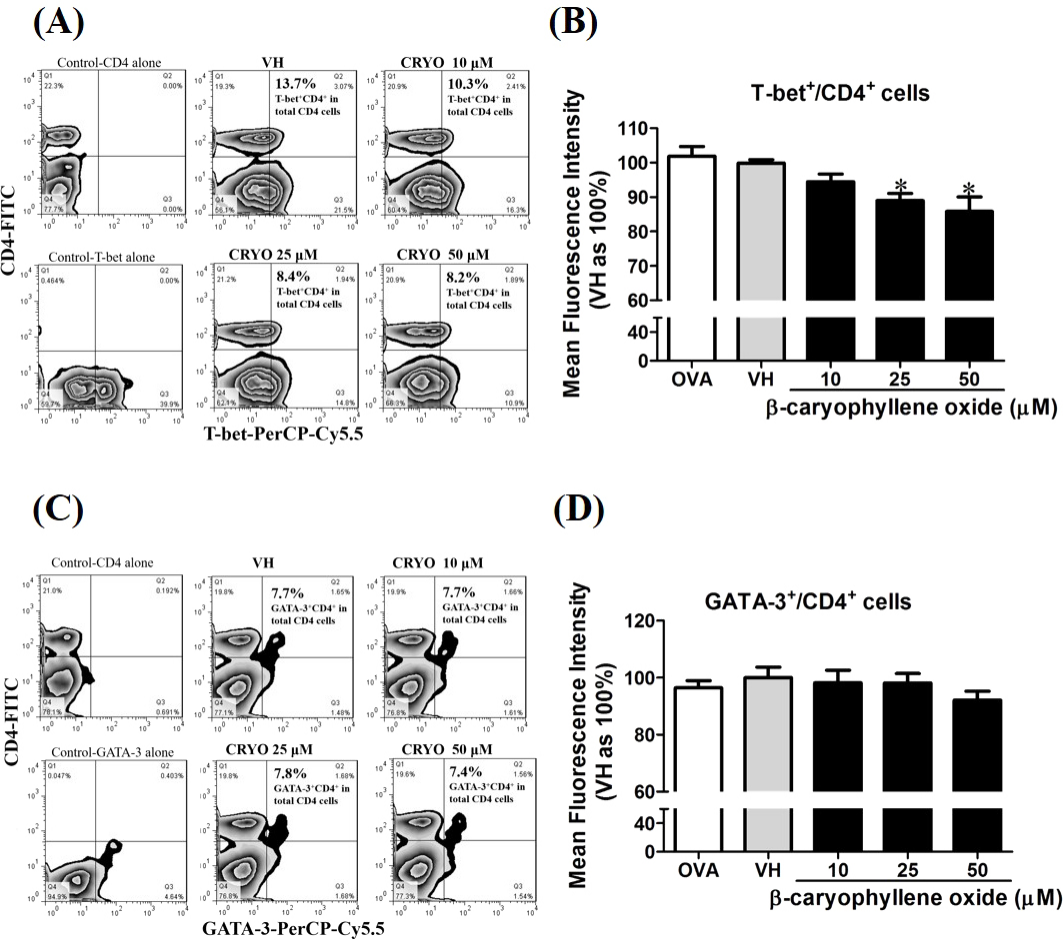
The effects of *β*-caryophyllene oxide on protein levels of T-bet and GATA-3 in CD4^+^ cells. After double staining of CD4 with T-bet or GATA-3 antibodies, tens of thousands of CD4^+^ cells were acquired on a BD LSR II flow cytometer. (A and C) The representative flow graphs show the cell population of T-bet, GATA-3 or CD4 cells and CD4^+^T-bet^+^and CD4^+^GATA-3^+^ double positive cells. The proportion of CD4^+^T-bet^+^ and CD4^+^GATA-3^+^in total CD4^+^ cells was quantified. (B and D) The mean fluorescence intensity (MFI) of T-bet or GATA-3 in total CD4^+^ cells was showed. The results are means ± SE of three separate experiments. *p < 0.05 was significant compared to the VH group.

To confirm the protein level of T-bet and GATA-3 in *β*-caryophyllene oxide-treated cells, intracellular staining of transcription factors in CD4^+^ cells were analyzed by flow cytometry. The proportion of T-bet^+^CD4^+^ and GATA-3^+^CD4^+^in total CD4^+^cells was quantified. Figure 12 showed that *β*-caryophyllene oxide decreased the total percentage of T-bet^+^CD4^+^ cells in CD4^+^ cells from 13% (VH) to 8% (*β*-caryophyllene oxide, 50 µM) and the level of mean fluorescence intensity of T-bet was significantly decreased (Fig. 12B) By contrast, the proportion of GATA-3^+^CD4^+^ and protein level of GATA-3 in CD4^+^ cells were not changed by *β*-caryophyllene oxide (Figs. 12C–12D).

## Discussion

In this presented study, we characterized the effects of the crude leaves extracts of *N*. *hiiranensis* and its selected secondary metabolites on T-cell functionality. Our results showed that the administration of the leaves extracts *in vivo* did not affect body weight, spleen index, cellularity, metabolic activity, nor the functionality of Th2 cells. By contrast, the *N. hiiranensis* leaves extract could modulate antigen-specific Th1 cell responses *in vivo.* The extracts effectively suppressed the production of antigenspecific OVA-specific IgM, IgG_2a_ and Th1 cytokines, including IL-2 and pro-inflammatory cytokine IFN-*γ* and IL-12. Furthermore, among the fourteen selected botanicals, *β*-caryophyllene oxide, hiiranlactone D, and *trans*-phytol inhibited IFN-*γ* cytokine production in a dose-dependent manner. In particular, *β*-caryophyllene oxide inhibited the mRNA expression of the transcription factor T-bet, IFN-*γ*, and IL-12R*β*2 which govern the development of Th1 cells in OVA-primed splenocytes. These results together demonstrated that *N. hiiranensis* and its secondary metabolites, especially *β*-caryophyllene oxide, could modulate antigen-specific T-cell responses via directly suppressed Th1 cytokine production and gene expression.

We demonstrated that crude extracts and secondary metabolites of leaves of *N. hiiranensis* possess suppressing antigen-specific Th1 cell-mediated immunity both *in vitro* and *in vivo*, suggesting that *N hiiranensis* may be a good source to isolate immunomodulatory botanicals for T cell-mediated immune disorders. IFN-*γ* plays an important role in the host defense against microbes and tumor cells. IFN-*γ* also has the ability to activate cytotoxic T cells, macrophages, and the killing activity of natural killer cells, as well as promote the production of opsonized antibodies to advance the phagocytosis of foreign antigens (Schroder et al., 2004; Street et al., 2002). However, overactive Th1 responses were associated with several immune diseases (Ito et al., 2006; Itoh et al., 2011; Rodgers & Miller, 2012). Robust production of IFN-*γ* has been shown to play indispensable roles in the initiation of dextran sodium sulphate-induced experimental inflammatory bowel disease in mice (Ito et al., 2006). In addition, mice with high levels of IFN-*γ* and IL-17 were more susceptible to induce symptoms of experimental autoimmune encephalomyelitis (EAE) through enhancement of Th1 cell-mediated immune responses (Li et al., 2014a). Based on the results, IFN-*γ* may play an initial role in several Th1-related immune diseases. Attenuation of IFN-*γ* production may provide a therapeutic strategy to management these immune disorders. Several natural compounds from medicinal plants have been shown to possess therapeutic potentials for Th1-associated diseases (Jeon et al., 2015; Wu et al., 2014). The extracts of mushroom *Phellinus igniarius* were demonstrated to relieve the symptoms of EAE through inhibition of IFN-*γ* production and lymphocyte proliferation (Li et al., 2014b). The extracts of *Brazilian propolis* were benefited to control the unbalanced cytokine networks of Th1 cells via suppressing the differentiation of Th1 cells and the generation of IFN*γ*-producing CD4 T cells in an autoimmune disease model (Okamoto et al., 2013).

Except for IFN-*γ*, Th1 cell development also involves the actions of both IFN-*γ* and IL-12. IL-12 plays an important role in the cellular immune responses by regulation of macrophage activation, promotion of Th1 cell growth and the differentiation of IFN-*γ*-producing Th1 cells in the host defense systems (Hamza, Barnett & Li, 2010). IL-12R*β*2, expressed on the activated T cells, is a heterodimeric receptor of IL-12 and acts as a key player in response to IL-12. T-bet response to IFN-*γ* may up-regulate IL-12R*β*2 surface expression and allow Th1 cell responsiveness to IL-12 (Afkarian et al., 2002). The opposite effects of IFN-*γ* and IL-4 on IL-12R*β*2 expression have been shown to involve in the commitment of Th1/Th2 differentiation (Hamza, Barnett & Li, 2010) For example, cannabinoids suppressed the IL-12 and IFN-*γ* production through inhibition of the IL-12R*β*2 while the production of IL-4 and expression of GATA3 were enhanced (Klein et al., 2004). On the other side, ribavirin induced T-cell differentiation and IFN-*γ* expression by upregulation of IL-12/IL-12R pathway (Shiina et al., 2004). In the present study, *β*-caryophyllene oxide significantly suppressed the expression of IFN-*γ*, T-bet, and IL-12R*β*2 suggesting that down-regulation of IL-12R*β*2 may be one of the underlying mechanisms.

Terpenoids were the second most abundant group of natural products widely distributed in the genus *Neolitsea*. They were also found in large amounts in the curry, cloves, cinnamon, black pepper, cannabis, guava, and moringa. Caryophyllene oxide is an FDA-approved food additive (Russo, 2011). This compound has been proved to have several biological activities. Caryophyllene oxide isolated from the leaves of the Jeju guava (*Psidium cattleianum*) have potent antitumor activities against several tumor cell lines with IC_50_ values of 4–28 µM (Jun et al., 2011). Caryophyllene oxide has also been demonstrated significant antidiabetic effects in streptozotocin (STZ)-induced diabetic rats (Basha & Sankaranarayanan, 2014). Caryophyllene oxide isolated from an unsaponified petroleum ether extract of the bark of *Annona squamosa* showed attenuated thermic stimulus-induced pain as well as carrageenan-induced paw edema in mice and rats at the doses of 12.5 and 25 mg/kg body weight, respectively. These data indicated the peripheral analgesic and anti-inflammatory activity of caryophyllene oxide (Chavan, Wakte & Shinde, 2010). In addition, it has been reported to reduce the mutagenicity of commonly discharged cigarette butts (Di Giacomo, Mazzanti & Di Sotto, 2015).

Hiiranlactone D, a unique secondary metabolite in the *N*. *hiiranensis* significantly suppressed IFN-*γ* production. Moreover, *trans*-phytol, also named phytol, differentially modulated the development of Th1 and Th2 cells by decreasing IFN-*γ* and increasing IL-4 production *in vitro*. *trans*-Phytol isolated from the stem of *Sinocalamus affinis* potently inhibited estrogen biosynthesis for the prevention and treatment of estrogen-dependent human cancer and may be a new source of tissue selective aromatase modulators (Guo et al., 2014). *trans*-Phytol identified in *Cajanus cajan* L. seeds, inhibited carrageenan-induced and decreased pro-inflammatory cytokine TNF-*α* and IL-6 *in vivo* and *in vitro* (Hassan et al., 2015). The commercial phytol reduced the number of contortions at doses of 25–200 mg/kg group in the acetic acid-induced writhing test *in vivo*. Moreover, this compound also showed a strong antioxidant effect to remove hydroxyl radicals and nitric oxide *in vitro*. These results demonstrated the pronounced antinociceptive and antioxidant properties of *trans*-phytol (Santos et al., 2013). Besides the above mentioned biological activities, there were no significant toxicity of phytol, including skin irritation, mucous membrane (eye) irritation, and mutagenicity. Phytol has already been used in cosmetics, household cleaners, detergents, and fragrance as an aromatic ingredient (McGinty, Letizia & Api, 2010). Interestingly, natural isoprenoid adjuvants, which is structurally similar to phytol, and phytol-derived compound PHIS-01 combined with the hapten, have been shown as the effective adjuvants on antibacterial immunity by increasing titers of IgG_2a_ antibody (Lim et al., 2006).

*β* −Caryophyllene oxide was the most potent immunomodulatory terpenoid examined in this study. Because *β*-caryophyllene oxide mainly inhibited Th1 cytokine, IL-2, and IFN-*γ* productions, we further investigated the underlying mechanisms of *β*-caryophyllene oxide on Th1 cell functionality. The mRNA expression of IFN-*γ* and two master transcription factors, T-bet, and Gata-3 which govern the differentiation of Th1 and Th2 cells, were studied. T-bet induces the production of IFN-*γ* and orchestrates the migration of Th1 cells, while GATA-3 induces the production of IL-4 and arranges the Th2 cell migratory program. *β*-Caryophyllene oxide significantly suppressed T-bet and IFN-*γ* expression, but the mRNA expression of GATA-3 and IL-4 was unaffected in the OVA-primed splenocytes. In addition, the expression of IL-12R*β*2 was decreased by *β*-caryophyllene oxide. Interestingly, IL-12, mainly produced by antigen-presenting cells, was not significantly altered suggesting the differential effects of *β*-caryophyllene oxide on activated Th1 cells. Although *β*-caryophyllene oxide at the high concentration slightly decreased IL-4 production, other Th2 cytokines IL-10 and IL-13 were unchanged. Collectively, these results demonstrated that *β*-caryophyllene oxide attenuated Th1 cell cytokine production via downregulation of IFN-*γ* expression, differentiation of Th1 cells, and activation of the IL-12R*β*2 pathway.

## Conclusion

The present study demonstrated that *N. hiiranensis*, an endemic *Neolitsea* in Taiwan, and its secondary metabolites have immunomodulatory activities. The leaves extract of the plant suppressed the antigen-specific IFN-*γ* production in OVA-sensitized mice, suggesting its potential immunomodulatory activities on Th1-skewed immune responses. Among the selected secondary metabolites, *β*-caryophyllene oxide was shown to effectively regulate IFN-*γ*, T-bet, and IL-12R*β*2 gene expression. In summary, *N. hiiranensis* and its terpenoids can regulate functionality and differentiation of Th1 cells and possess potential as therapeutic agents for Th1-mediated immune disorders.

##  Supplemental Information

10.7717/peerj.2758/supp-1 Figure S1 Supplemental data-IFN-r production in CD4 T cells ***β*-caryophyllene oxide attenuated IFN-***γ* **production in CD4^+^** **T cells.** The splenocytes were treated with *β*-caryophyllene oxide (10–25 μM) for 36 h and then labeled with anti-CD4^+^ and anti-IFN-*γ* ^+^ mAb. (A) The representative dot plot showed the distribution of CD4^+^IFN-*γ*^+^ cells. (B) The representative histogram of IFN-*γ* in total CD4^+^ cells. (C) The mean fluorescence intensity of IFN-*γ* in total CD4^+^cells was showed. The results are means ± SE of three separate experiments evaluated using flow cytometry.*p<0.05 was significant compared to the VH group

10.7717/peerj.2758/supp-2 Data S1 *In vivo* data

10.7717/peerj.2758/supp-3 Supplemental Information 1 14 compounds *in vitro*

10.7717/peerj.2758/supp-4 Supplemental Information 2 *In vitro* cytokine

10.7717/peerj.2758/supp-5 Supplemental Information 3 QPCR

## Additional Information and Declarations

### Data Availability

The following information was supplied regarding data availability: The raw data has been supplied as a Supplemental File.
