# Supplementary figures and images for: Attenuation of antigen-specific T helper 1 immunity by Neolitsea hiiranensis and its derived terpenoids
Source: PeerJ. 2016 Dec 7;4:e2758. doi: 10.7717/peerj.2758 (PMC5363408; doi:10.7717/peerj.2758)

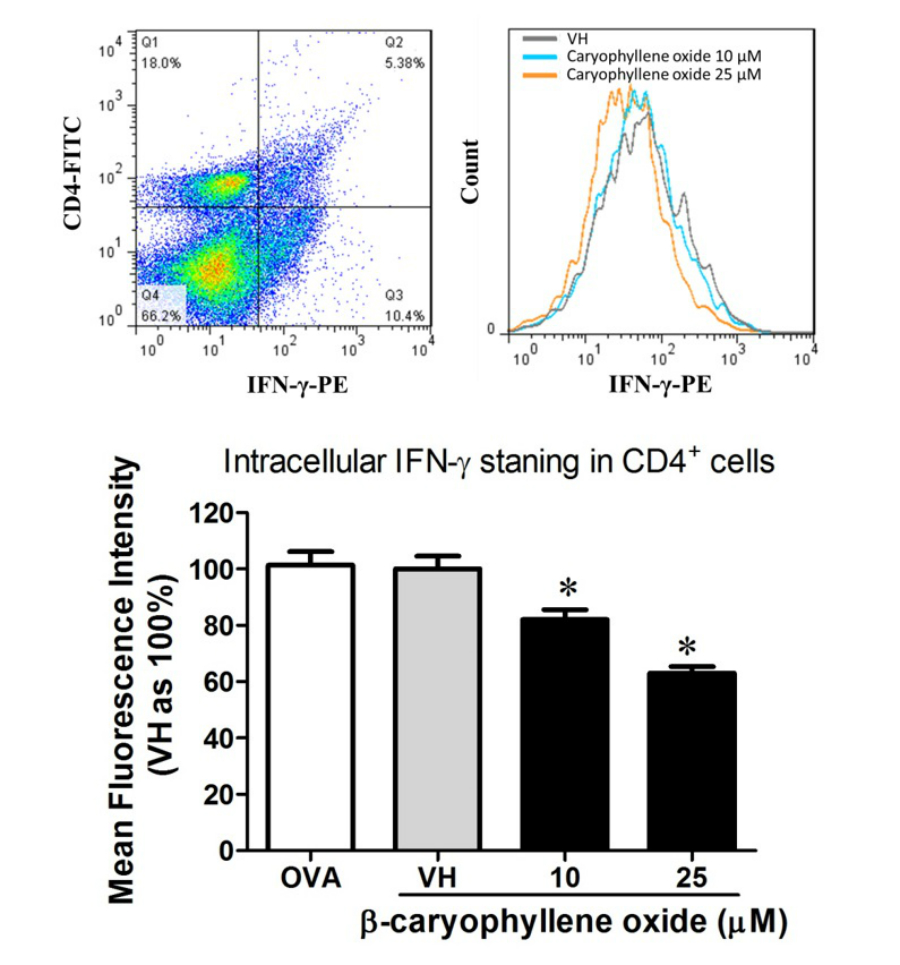

Supplement: Figure S1 — β-caryophyllene oxide attenuated IFN-γ production in CD4+ T cells. The splenocytes were treated with β-caryophyllene oxide (10–25 μM) for 36 h and then labeled with anti-CD4+ and anti-IFN-γ + mAb. (A) The representative dot plot showed the distribution of CD4+IFN-γ+ cells. (B) The representative histogram of IFN-γ in total CD4+ cells. (C) The mean fluorescence intensity of IFN-γ in total CD4+cells was showed. The results are means ± SE of three separate experiments evaluated using flow cytometry.*p<0.05 was significant compared to the VH group [file peerj-04-2758-s001.jpg]
